# The impact of Nursing and Midwifery Council revalidation on the professional identity of academic staff in a higher education institution: A qualitative study

**DOI:** 10.1002/nop2.224

**Published:** 2018-11-28

**Authors:** Julie Attenborough, Stephen Abbott

**Affiliations:** ^1^ School of Health Sciences City, University of London London UK

**Keywords:** academic identity, midwifery, nursing, revalidation

## Abstract

**Aims:**

To explore Nursing and Midwifery Council (NMC) revalidation as a process experienced by nursing and midwifery academics and its impact on their sense of professional identity.

**Background:**

The introduction of revalidation nurses and midwives in the UK in 2016 caused some anxiety amongst registrants in higher education.

**Design:**

A qualitative study using a purposeful sample involving thematic analysis of semi‐structured interviews with academic staff.

**Methods:**

Ten registrants completed a semi‐structured interview in a higher education institution.

**Results/Findings:**

Clinical credibility: participants were self‐conscious about time away from practice but retained strong links with clinical settings reviewing evidence and reports of current practice. The revalidation process: staff were generally positive about NMC revalidation. Professional identity: participants identified as nurses and midwives first and academics second.

**Conclusions:**

The findings replicate previous studies about professional identity among healthcare professionals in higher education; this study reports the contribution of revalidation amongst nurses and midwives in higher education institutions.

## 1 INTRODUCTION

Recruitment and retention of academic staff in nursing and midwifery is of international concern, linked to the global shortage of nurses (Laurencelle, 2016). Revalidation was introduced in the United Kingdom by the Nursing and Midwifery Council (NMC) in April 2016 in response to a recommendation of the report by Sir Robert Francis QC (2013) into the role of commissioning, supervisory and regulatory bodies in the monitoring of standards at Mid Staffordshire Foundation NHS Trust. Revalidation replaced the system of post‐registration education and practice (PREP) and aims to promote safe and effective practice, with greater accountability and emphasis on professional development (Lanlehin, 2018a, 2018b). The process involves demonstrating competence and adherence to the Code (Nursing & Midwifery Council, 2018), currency, reflection and engagement with professional networks within a defined area of practice. Full details of the revalidation process in the UK are available (http://revalidation.nmc.org.uk/).

The NMC reported 2% of registrants revalidating in the first and second years of the process as working in education, with 1.1% employed by universities (NMC, 2017; Nursing & Midwifery Council, 2018). It is therefore not surprising that the emphasis in the implementation of revalidation was on nurses and midwives employed in clinical practice.

As a new process, some challenges emerged in implementing revalidation in a higher education context; the most common was academic staff anxiety about the strength or weakness of their identity as a nurse of midwife in relation to their lack of current clinical practice and how this might affect their clinical credibility (Attenborough, 2017). The NMC revalidation process could therefore be seen as eroding educators’ sense of identity as a nurse or midwife by confronting them with the limitations in their clinical experience and expertise; or conversely, to enhance that sense of identity by affirming the continuity of experience and expertise across the clinical/educator boundary.

The overall aim of this study was therefore to explore the impact of the NMC revalidation process on the professional identities (as clinician or educator or both) of academic staff at City, University of London. It is anticipated that the results of the study will inform future development of nursing and midwifery as academic subjects and the progression of academic registrants in the academy.

## 2 BACKGROUND LITERATURE

In the United Kingdom, nursing and midwifery courses have been part of universities' educational offering for more than 20 years, yet the debate about their position in higher education continues, both inside and outside the academy (Andrew, 2012; Gillett, 2014; Oliver, 2017; Thompson & Clark, 2017). The self‐perception of professional identity in nursing and midwifery academics and the tension between their roles, as educator, researcher and clinician have been explored in several studies (Andrew, 2012; Andrew and Robb, 2011; Andrew et al., 2014; Lopes, Boyd, Andrew, & Pereira, 2013). The issue of identity came back into sharp focus in universities when NMC revalidation was introduced. Although the revalidation process enables academic staff to define their area of practice as education, some registrants asked to be released from the university to undertake clinical practice to revalidate (Attenborough, 2017). It is notable that in the UK registrants cannot undertake return to practice or registration from overseas without undertaking a clinical assessment, or clinical practice, there is no education route. In the United States, the National League for Nursing recognizes the academic nurse educator as an advanced practice role (Booth, Emerson, Hackney, & Souter, 2016).

The desire of nurses, midwives and allied health professionals in academia to maintain their clinical identities is widely reported in the literature (Findlow, 2012; Laurencelle, Scanlan, & Brett, 2016; Murray, Stanley, & Wright, 2014; Smith & Boyd, 2012). The reluctance to take on the new identity of researcher or lecturer has been attributed to lack of confidence and institutional support to undertake the new role (Andrew, Lopes, Pereira, & Lima, 2014). Other papers contrast the positive image of a caring and compassionate nurse in clinical practice with the negative image of educators and education (Andrew, 2012; Gillett, 2014; Oliver, 2017). Duffy (2013) describes three different identities adopted by nurses entering academia, the most positive for integration into the academy being a “hybrid identity” (p623), incorporating practice, academic development and a clinical identity.

A “duality of professional practice in nursing” is identified in one paper, with academic staff describing workload in terms of teaching and maintenance of clinical credibility, with less regard for research and scholarly activity, despite the recognized need to develop a research base for nursing (Andrew & Robb, 2011, p. 429). Lopes et al. (2013) discussed similar findings in a Portuguese study, as did Logan, Gallimore, and Jordan (2016) in a study of transition from clinician to academic in Australia and the UK. Andrew, Ferguson, Wilkie, Corcoran, and Simpson (2009) note the amount of clinical expertise and contact required by nurse educators along with the requirement to build an academic profile, as students require support in clinical practice and the minimum number of hours in programmes are prescribed by the regulator; alongside the tendency (at least initially) to import the teaching pattern of the traditional hospital‐based training school into higher education. Andrew observes that the continuing debate about the legitimacy of nursing as an academic subject also raises the issue about whether nurse academics are contributing to the knowledge‐base on equal terms, which may also affect their identity as academics.

Logan et al. (2016) reported that lecturers in nursing feel conflicted in their multifaceted, multiple‐identity role with the necessity to establish credibility in teaching, research and administration, whilst also maintaining credibility in clinical practice. Approaches to identity exploration include the internal psychological approach taken by psychologists and the societal approach by sociologists (Côté & Schwartz, 2002) The professional identity of nursing and midwifery registrants in higher education includes elements of both and links to self‐esteem, the esteem of the profession and the individual's learning through personal experience. Nursing and midwives’ identity is influenced by societal images and lack of visibility, along with their internal self‐concept (Hoeve, Jansen, & Roodbol, 2014).

The need to be credible in teaching and research can conflict with the belief in the central importance of patient care and outcomes (Murray et al., 2014), while the focus of nursing on “caring” makes it difficult for nurses to identify as academics (Duffy, 2013). Smith and Boyd (2012) report grief and a sense of loss of role and credibility, along with the experience of going from the top of one profession to the bottom of another with the associated loss of status and confidence. The feeling of loss and the impact on identity and self‐worth, along with a perceived lack of appreciation of professional values is reported by Duffy (2013). Andrew (2012) discusses guilt in relation to leaving practice and the difficulty in coping with the subsequent reduction in clinical expertise.

In a qualitative meta‐synthesis investigating the transition from clinical practice to academia for nurses and allied health professionals, Murray et al. (2014) identified four phases of evolving into an academic lasting between 1‐3 years. Phase one where lecturers most rely on their identity as clinicians, whilst phase two includes the devaluing of their clinical experience in favour of doctorates, research income and publications. In phase two, lecturers also spend a disproportionate amount of time preparing for teaching at the expense of research and scholarly activity. Phase three involves an adjustment to a less student‐centred/patient‐centred way of working, with more self‐direction and less task‐based activity. Phase four is described as “evolving into an Academic” (Murray et al., 2014, p. 393), with problems relating to professional identity (clinician or academic). There is some evidence that this relates to feeling of unworthiness in the academic role as described elsewhere in the literature (Farnworth, Rodger, Curtin, Brown, & Hunt, 2010), while Smith (2010) describes a process of academic socialization, which for some is difficult and perplexing; and a requirement for resilience and determination to survive and succeed.

## 3 THE STUDY

### 3.1 Aims

The study aimed to explore academic staff's experiences of NMC revalidation and its impact on their professional identity as nurse or midwife educator in a higher education institution. The study investigated the relationship between NMC revalidation and academic staff perception of themselves as registrants. As it was undertaken shortly after the introduction of the new revalidation process, the study also sought to identify any technical and implementation issues in establishing the process in a university.

### 3.2 Design

The project adopted a qualitative approach, examining the experiences and feelings of staff about revalidation via a semi‐structured interview. The focus of the semi‐structured interviews was to examine the lived experience of the process of revalidation, including prior expectations, actual experiences and later reflections. In particular, because some had expected to have to return to clinical practice to revalidate (Attenborough, 2017), interviewees were asked about their sense of identity as NMC registrants employed in an academic role. Interviews were audio‐recorded and transcribed.

### 3.3 Sample/participants

All nurse and midwifery registrants working in academic roles in the School of Health Sciences were identified through the human resources department at the university. An online survey about preparation for revalidation was distributed to all registrants in an academic role at the university. This survey was for administrative purposes only and was not part of the research. However, at the end of the survey, registrants were asked if they would be prepared to take part in a semi‐structured interview with an independent researcher. A maximum variation sample was drawn from those agreeing, reflecting diversity of specialism (nursing, midwifery, health visiting) and length of time since working in a clinical role. The study comprised 10 interviews, the number reflecting available resources.

### 3.4 Data collection

Data were collected using the following topic guide.

Question areas:

1. Introduction and establish which professional area the member of staff predominantly teaches in: nursing, midwifery, SCPHN, none of these.
2. When were you last employed in clinical practice? What was your job/role?
3. Can you tell me about your experience of and/or knowledge of NMC revalidation (some staff will have revalidated already, some will not).
4. What are your views on the introduction of revalidation for all nurses, midwives and SCPHN?
5. How did you feel when you first heard that academic staff would need to revalidate with evidence of their practice, CPD and feedback?
6. To what extent do you feel that your professional identity has been affected by the experience of revalidation?
7. What support did you get to revalidate from City; did you get support from elsewhere?
8. Do you have fellowship/senior fellowship/principal fellowship of the Higher Education Academy (HEA)? How do you view this professional recognition in higher education in relation to your NMC registration?
9. Would you recommend an academic career in nursing/midwifery/SCPHN to somebody working in clinical practice currently?

Probe areas included:

- How academic staff perceived their clinical credibility in the eyes of students
- Specific examples of feeling a lack of credibility when teaching or interacting with students Connectedness to the profession of nursing or midwifery whilst working in an academic role.
- Specific examples of connectedness to the profession and professional identity relating to NMC revalidation.

This semi‐structured interview schedule was devised to contain only open‐ended, non‐directive questions to encourage free narrative and detailed responses (Eatough & Smith, 2017). The topic guide formed a basis for a conversation; it was not intended to be prescriptive and certainly not limiting to the expressed experience of the participant. It was intended that the interviewee takes the lead during the conversation (Biggerstaff & Thompson, 2008) allowing them to express their thoughts, understanding and experience and the interviewer frequently paraphrased and reordered the questions to reflect the conversational direction taken by the interviewee.

The second author conducted the interviews, which took place in a convenient location for the participants, generally in their workplace but in a private room. Apart from the researcher and the participant no one else was present for the interviews, the interviews were all conducted on a one‐to‐one basis. No one recruited into the purposeful sample dropped out of the study. The interviewer is an experienced qualitative researcher with no direct working relationship with the participants and no experience of revalidation as he is not a nurse or midwife.

### 3.5 Ethical considerations

Ethical approval was granted by the School of Sciences research ethics committee. As the Principal Investigator was in a senior position in the School and sits on the school executive, it was therefore very important that participants were assured of anonymity and that interviews were conducted by an independent researcher who was not connected to their everyday work life. Participants were advised that participation was entirely voluntary and that they could withdraw at any time without providing a reason. Staff who volunteered to participate in the study but did not wish to complete it were assured that this would not affect their employment in any way; this was clearly stated in the Participant Information Sheet (PIS) and consent form.

Permission was sought for recording of interviews and consent forms were completed for both the online survey and the semi‐structured interviews. Participants who volunteered for interview were contacted by an independent researcher.

All analysis was conducted anonymously.

### 3.6 Data analysis

A team data analysis approach was adopted (Crist & Tanner, 2003): two independent researchers analysed the data separately and discussed results to ensure a consistent approach and rigorous and valid results. Interview transcripts were analysed, with a deliberate search for divergent cases. Themes were inductively derived from the data, although both analysts were aware of the topic guide and of the aims of the study and this will have influenced their perception of emerging themes. In addition, a qualitative research data expert reviewed and confirmed the identified themes, which were three, as follows:

- Clinical credibility
- The revalidation process
- Professional identity.

## 4 FINDINGS

Seventy‐six registrants were employed as academic staff at the time of the study, of whom forty‐three (57%) responded to the survey. About 22 of those were willing to be interviewed and a maximum variation sample of 10 was drawn, as set out in the Table 1. All had undergone the revalidation process.

**Table 1:** Staff interviewed

Number	Lecturer/researcher in:	Last in practice	Higher Education Academy fellow?
L1	Health visiting	2012	Y
L2	Adult nursing	1992 approx.	Y
L3	Midwifery	2016	N
L4	Adult nursing	2003	N
L5	School nursing	2014	Y
L6	Midwifery	2013	Y
L7	Mental health nursing	2014	N
L8	Children's nursing	1998	N
L9	Mental health nursing	1997	N
L10	Children's nursing	2012	Y

### 4.1 Clinical credibility

By clinical credibility, we mean lecturers’ credibility as clinicians in the eyes of students. Those interviewed tended to speak at some length about their clinical credibility and the risk that it could be reduced over time. Most felt fairly confident that their own credibility was sufficient for their role in the university. This was often because they visited health care settings regularly as part of their role:

I very much spend time talking to clinical staff and to students. So I update what’s going on out there all the time, I understand what their issues are. (L4)

One still worked in the clinical area with which the lecturer was linked, but another argued that working on a ward would be less effective in enhancing credibility than was keeping up to date with current research, which was necessary for teaching:

I have time to read far more and think far more now, about midwifery research and midwifery knowledge, than I certainly would have done clinically. (L6)

This person also pointed out that specialism in practice restricted broader knowledge: for example, an ante‐natal midwife might know relatively little about breast‐feeding.

Others referred to their collaboration with healthcare providers on projects, committees and training. A small number taught clinical skills using simulation and therefore needed to ensure that they were up to date.

The point was also made that it was unusual that the basics of care in a particular clinical area underwent radical change, so lecturers felt largely up to date. They sometimes realized in talking with students that there was an innovation that they needed to find out about. No‐one reported feeling uncomfortable that students had been aware of lecturers’ ignorance, though a few did acknowledge some loss of credibility in their own eyes. One believed that students did prefer being taught by people who were visibly hands on:

being much more up to date literature wise … is not valued as much as, have you got the manual dexterity if you’re whipping down NG tubes (L8)

Another wondered what students were thinking but chose not to articulate. The midwifery lecturer added:

A lot of students say, ‘Do you still deliver babies?’ … they do ask that question… (L6)

Credibility issues were affected by the topics being taught: a registrant who taught biology, for example, felt that if the lecturer made an error in referring to clinical practice, this was insignificant given the subject of the lecture. Another reported:

moving away from teaching the clinical skills to more generic skills that possibly don’t need me to be absolutely up to date with the way things are done … it’s made me feel more comfortable with what I’m teaching. (L1)

### 4.2 Revalidation

All of those interviewed had undergone the revalidation process. Most had found it relatively straightforward and had attended workshops provided by the university. A few mentioned the revalidation file provided by the university, which had helped them to organize material easily. The workshops had dispelled quite widespread fears that lecturers would have to go back into practice to acquire clinical hours. Several also said that the materials provided online by the NMC were straightforward and helpful. Some who were less positive about the process, explained that this was due to their own procrastination or disorganization.

Informants were asked if they could remember how they felt when they first heard about revalidation. Most recalled assuming that it would be another meaningless bureaucratic chore and the process had been a pleasant surprise. It was useful to look back in a structured way and, for example, to have an overview of all the training attended:

The biggest benefit was that I had to think about what I’d done, why I’d done it, what I’d learnt. And I wouldn’t necessarily have done that in such a structured way if I hadn’t had to go through the process. (L1)

More often, though, it was the need to reflect and to discuss reflections that had been useful:

It made me genuinely reflect a lot on what I had done and what I had learnt and how it had changed some of my practices and my teaching. And I would actually recommend it to people as a good thing to do. (L9)

Generally, informants considered the process an improvement on PREP:

Before, we were supposed to do something called PREP and I don't think people did that very much … [now] it's for us to prove, provide evidence that we're competent to be nurses. Given the numbers, I think it's reasonably rigorous... I don't know how else you can do it really. (L5)

As this quotation implies, however, people saw the process as having limited robustness. A few considered the NMC should be asking to see the revalidation materials of at least a certain number of registrants (which does happen in practice). Most people reported that they and their colleagues were taking revalidation seriously, though one reported rumours of dishonesty elsewhere. Another was concerned about the requirement to provide feedback:

We self‐ select our own feedback to include in our revalidation … clearly you are going to select all positive feedback … I think that it would be more useful to include your yearly appraisals for the last three years… it would demonstrate an on‐going progression rather than snippets of time. (L10)

Only one person had no faith in the process and saw it as a poor substitute for conscientious management by employees:

I just feel it's a paper and pencil exercise. I am not convinced that it will actually do anything for the profession… The real problem I think is in practice and it's with the NHS organisations who manage those practitioners. (L4)

Several informants described how the revalidation process had to a certain extent strengthened their sense of connection with the profession:

When I chat to nurses, sometimes they say to me, ‘Oh, when’s your revalidation due up?’ and it’s something we all share. (L2)

It reaffirmed that I do agree with the values that underpin the profession. It made me think about how I've interpreted them and what I've done with them. … It does make you think about what is it about nursing that I value. (L1)

Others felt very connected to the profession and were very familiar with the NMC Code, because of the topics they taught, such as accountability.

Some sympathy was expressed for registrants in practice who might find it harder:

I know for quite a few nurses this is going to be a problem… Structurally, I don't think the employers are good at providing time, space, support for nurses to maintain their education. (L9)

### 4.3 Professional identity

Most of those interviewed spoke of being primarily a nurse or midwife:

I relate to being a nurse first and foremost and the idea of being a lecturer, yes, that's a difficult one … Being a lecturer in nursing doesn't feel like being a proper lecturer because it's more about, you got there on the merit of being a nurse or a health visitor or whatever, rather than, you got there on the merit of being an academic. (L1)

I tell myself I’m a nurse who teaches, rather than a teacher who nurses. (L8)

They did not regret their academic role and no‐one would hesitate to recommend an academic career to those who were interested and suitable.

Lecturers were asked how connected they felt to their profession. Most still saw themselves as a healthcare professional first and foremost. For some, this was in part because they had been a nurse for much longer than they had been a lecturer (see Table 1). Others recognized a strong emotional connection:

I do truly believe that there is something special about nursing and I partake in that, I sign up to that … I worry that sometimes it's a bit of a sentimental… it's part of my identity, but I'm not absolutely sure how much, how real it is. (L2)

Another reported that, when she had had the opportunity to move abroad:

the thought of losing the nursing element of my identity stopped me from going. (L1)

Another pointed out that their own knowledge of hospital care was greater now than it had been when in practice in the community.

One person made explicit what was implied by all the others: that nursing and midwifery are disciplines that include a wide range of activities and so can embrace non‐clinical roles:

Nursing is so many different things that you can be....you know....a community nurse, a hospital nurse, a hospice nurse, a clinical nurse specialist, a manager, a teacher, a research nurse... So it is just a different way that you interpret your role as a nurse. (L10)

There was little evidence that the educator identity was of primary importance and even the four staff who were fellows of the HEA appeared to think that that fellowship was not significant nor particularly beneficial:

I have nothing to do with the HEA, I have never used their resources, I don’t quite know what they’re there for … (L6)

One of these felt that the process of becoming a fellow was:

more of a tick box exercise, to be honest … it is important in terms of making sure that we maintain ongoing development in terms of being a teacher as well as a nurse. But I am not entirely sure that a one off application to the Higher Education Academy is the way that that is achieved. (L10)

However, two who were not yet fellows saw the HEA more positively as lending academic credibility:

I would like to apply to be a senior fellow …I think it's very important… I want recognition of excellence in teaching… (L7)

## 5 DISCUSSION

The issue of clinical credibility, though considered important by lecturers was not thought to be achieved only through direct clinical practice; academic staff appreciated the need to be credible in several domains, such as maintaining their registration through revalidation, being up‐to‐date with the latest evidence and research and acquiring academic skills and competencies; this is consistent with findings elsewhere (Andrew et al., 2009; Andrew, 2011; Logan et al., 2016). Lecturers in this study expressed a need to maintain clinical credibility; perceiving students as highly valuing it. This contrasts with a study examining the value of being taught by registrants to students (Attenborough & Abbott, 2018), where students reported it not being important if lecturers were not absolutely up‐to‐date with every detail of practice. Andrew (2011) suggests a “close to practice culture” (p430) where actual practice is not possible, whereas Baldwin, Mills, Birks, and Budden (2017) differentiate between clinical currency and clinical legitimacy, clinical credibility and the currency of nursing knowledge, acknowledging the importance of role‐modelling and “storytelling” (p2) of lecturers’ professional and clinical lives. Significantly, in *Midwifery 2020: Delivering Expectations, *the Department of Health called for innovative solutions to enable midwifery academics employed in universities to maintain their clinical credibility: “Midwifery lecturing staff who are credible in the practice environment are well positioned to support students....” (Department of Health, 2010, p. 41).

However, clinical credibility and identity were quite distinct; most participants identified first as nurses or midwives, this being a very important part of their professional identity. This is widely reported in the literature but may be detrimental to progression in a higher education institution, with staff prioritising teaching, support for students in clinical practice and pastoral care for students over gaining a doctorate or research (Duffy, 2013; Jackson, Peters, Andrew, Salamonson, & Halcomb, 2011; Smith & Boyd, 2012). This identity related to how long they had been out of practice and replicates the findings of Murray et al. (2014). Interestingly, the process of revalidation led to increased connectedness with colleagues in clinical practice. Whether this is detrimental to the establishment of nursing and midwifery as academic subjects is debatable and may relate to the type of higher education institution participants are located in (Lopes et al., 2013), older research‐intensive universities having different expectations to newer teaching‐intensive institutions.

Anxiety about the revalidation process in relation to clinical competence was articulated to a small extent and interviewees explained how workshops provided by the university had reassured them (Attenborough, 2017). Most participants were positive about the process of revalidation, particularly valuing the impact of the reflective discussions and CPD recording. The relationship between the HEA fellowship and NMC registration was complex, with those who had achieved fellowship of the HEA appearing not to value it especially and certainly not considering it as part of their identity. For those who aspired to fellowship the response was far more positive. Those with fellowship experienced it as a tick‐box procedure, not especially linked to their role, which was untrue for NMC registration, which may demonstrate a lack of engagement with the HEA, especially with the community of practice that it supports. In another study, however, Fisher et al. (2018) reported the perception among some registrants of NMC revalidation being a “tick‐box exercise” (p9) that lacked the robustness to inspire public confidence.

There was some evidence of academic staff considering that they had been appointed to an academic role because of their clinical qualification, rather than academic or research qualities (Farnworth et al., 2010), confirming the perception reported by Duffy (2013) that in some ways nursing does not belong in higher education and that nurse academics felt nursing was not held in such esteem as other subjects in (Findlow, 2012). Furthermore, Lanlehin (2018a, 2018b) identifies a gap in structured support for those recruited from clinical practice, curtailing progression in the academy.

Interviewees were positive about an academic career and did not express regret about their choice. This is an interesting finding given their identity as nurses and midwives and may relate to the work of Laurencelle et al. (2016), whose subjects explained their motivation to work in higher education as a desire to teach, considering teaching as part of nursing practice and wanting to educate the next generation (Smith & Boyd, 2012; Weidman, 2013).

### 5.1 Limitations

Because of limited information about those not participating, it is not possible to define any sampling bias. The study took place in a pre‐92 UK university, the experience of lecturers in nursing and midwifery may be different in a post‐92 UK university, reflecting the research‐teaching nexus as suggested by Lopes et al. (2013) (most nursing and midwifery lecturers in the UK are employed in post‐92 universities). It would be useful to test our results with larger and more diverse samples of academic nurses and midwives.

The invitation to take part in the research was sent by an independent researcher to reduce pressure to participate and further ensure confidentiality. However, the PIS clearly stated that the principal investigator is a senior member of academic staff in the school, which may have inhibited some of those interviewed, or deterred some from volunteering.

## 6 CONCLUSIONS

Lecturers delivering nursing and midwifery courses in the higher education institution had a favourable view of NMC revalidation. Participants identified strongly as nurses and midwives rather than academics. Lecturers reported some self‐consciousness about lack of clinical currency but were clear about the ways they kept their practice current.

The system of revalidation was thought to work well, though some participants questioned its robustness and saw it as more bureaucratic than developmental and assuring.

Staff valued their registration with the NMC more highly than fellowship of the HEA, but some perceived that the university valued fellowship more highly.

### 6.1 Recommendations for further research

At the time of the study, NMC revalidation had been in place for 1 year. The role of revalidation in contributing to the identity of lecturers working in higher education and the relationship between this and accepting and integrating registrants into the academy is an important area deserving of further investigation as the process embeds over time, with larger and more diverse academic participants. Additionally, there is paucity of data about the impact of revalidation on students as the registrants of the future and future studies should address this. Furthermore, the finding that the process of revalidation made registrants feel more connected with practice (and especially with practice colleagues) is an important one, worthy of further study as revalidation for nurses and midwives in the United Kingdom is fully embedded.

## CONFLICT OF INTEREST

No conflict of interest has been declared by the authors.

## IMPACT STATEMENT

This study investigated the impact of professional revalidation on the professional identity of nurses and midwives in academic roles. The process of revalidation reinforced identity as nurses and midwives first and academics second and has implications for progression of registrants within the academy. Recruitment and retention of academic staff in nursing and midwifery is an international concern, with a world‐wide shortage of registrants and educators. Higher Education Institutions should consider how they can support registrants to succeed in the academy with a career structure that recognizes their dual identity and professional registration requirements, and demonstrates appreciation for nursing and midwifery as academic subjects.
